# When to Consider Risk-Reducing Mastectomy in *BRCA1/BRCA2* Mutation Carriers with Advanced Stage Ovarian Cancer: a Case Study Illustrating the Genetic Counseling Challenges

**DOI:** 10.1007/s10897-017-0136-1

**Published:** 2017-08-05

**Authors:** Beverley Speight, Marc Tischkowitz

**Affiliations:** 1 East Anglian Medical Genetics Service, Cambridge Biomedical Campus, Box 134, Level 6, Addenbrooke’s Treatment Centre, Addenbrooke’s Hospital, Cambridge, CB2 0QQ UK; 2 Academic Department of Medical Genetics, University of Cambridge, Cambridge, UK

**Keywords:** BRCA1/BRCA2, Ovarian cancer, Risk-reducing breast surgery, Genetic counseling

## Abstract

Germline mutations in *BRCA1/BRCA2* significantly increase the risk of breast and ovarian cancer in women. This case report describes a *BRCA1* germline mutation identified in a woman with stage IV epithelial ovarian cancer and the provision of genetic counseling about *BRCA1*-associated breast cancer risk in the three years following diagnosis. The report centers on the patient’s enquiry about risk-reducing breast surgery. We focus on the challenges for health professionals and patients in understanding and balancing the risks and benefits of major prophylactic surgery in the context of a potentially life-limiting cancer diagnosis. Breast cancer risk management in *BRCA1/BRCA2* carriers with advanced ovarian cancer is an under-explored area of genetic counseling research. This article includes a case report, a review of the relevant literature and considers some implications for practice.

## Introduction

*BRCA1/BRCA2*-associated cancer risk management includes increased cancer surveillance and risk reduction strategies. For breast cancer risk, additional screening using mammography and MRI is recommended with the aim of detecting cancer at an early stage (Kriege et al. 2004). The most significant breast cancer risk reduction strategy involves bilateral mastectomy, usually combined with breast reconstruction. Risk reducing surgery is usually most relevant between approximately ages 30–60, during the years when relative breast cancer risk is high and the risk-benefit balance is more likely to be favourable. In countries with *BRCA1* and *BRCA2* management guidelines, discussion with women on the risks as well as the potential benefits associated with surgery is recommended (Easton et al. 2015). A woman’s age and general health have a critical impact on these risks.

Risk reduction strategies are most relevant to women identified as carrying a *BRCA1* or *BRCA2* mutation who have not yet developed cancer and the role of risk-reducing surgery in the management of hereditary breast and ovarian cancer is already well established (Hartmann and Lindor 2015). However, identification of a hereditary *BRCA1*/*BRCA2* mutation in a family is often via initial diagnostic testing in an individual with cancer. The benefits of identifying a germline mutation soon after diagnosis can have implications for early treatment decisions and access to more effective therapies, e.g. poly(adenosine diphosphate ribose) polymerase (PARP) inhibitors (Konecny and Kristeleit 2016).

In the UK, as in other countries, there is movement towards offering more women with non-mucinous epithelial ovarian cancer genetic testing of *BRCA1* and *BRCA2*, irrespective of family history (George 2015). In our center, genetic testing of *BRCA1* and *BRCA2* is automatically offered to every woman with epithelial ovarian cancer diagnosed below age 70 years. This is based on research showing that 12% of women diagnosed below age 70 years in our region have a germline mutation, without taking family history into account (Plaskocinska et al. 2016).

This type of unselected genetic testing in newly diagnosed women with epithelial ovarian cancer is leading to more families without a strong history of breast cancer being found with germline *BRCA1/BRCA2* mutations. In these families, there may not have been any prior anticipation of an increased breast cancer risk, given the absence of any family history of breast cancer. Women with ovarian cancer in whom the pathogenic variant has been identified may be adjusting to information about a high breast cancer risk for themselves and other family members at a vulnerable time, if this comes shortly after an ovarian cancer diagnosis.

There is evidence that genetic testing soon after ovarian cancer diagnosis does not add to the negative psychological response caused by the cancer diagnosis itself, but that finding out about *BRCA1/BRCA2* carrier status may lead to a slight increase in the psychological burden at that time (Plaskocinska et al. 2016). However, little is known about how women identified as carrying a *BRCA1/BRCA2* mutation after an ovarian cancer diagnosis adjust to and view their breast cancer risk. The experience within our clinical genetics service is that most women accept the offer of referral for increased breast surveillance and few request detailed information on risk-reducing breast surgery. Risk reducing surgery is generally not considered as beneficial in the initial years after stage III or IV ovarian cancer diagnosis, due to the focus on treatment, uncertainty about recurrence and prognosis. However, breast cancer risk reduction is a valid issue for women to raise and awareness of risk-reducing surgery has increased in recent years due to high-profile publicity (Evans et al. 2014). Information about risk-reducing breast surgery is also provided by charities aimed at supporting and informing women after an ovarian cancer diagnosis. Increased awareness of risk reducing surgical options is likely to increase the number of women at high risk of breast cancer asking about the potential benefits this can bring and there is some evidence of this happening in recent years (Evans et al. 2015).

## Case Report: Background

This case describes a 54 year old diagnosed with stage IV high grade serous ovarian cancer. The patient provided adequate family history information, reporting relatively few cancers in a large family, except for suspected ovarian cancer in her paternal grandmother (unconfirmed). Genetic testing was carried out via the Genetic Testing in Epithelial Ovarian Cancer study (Plaskocinska et al. 2016). This study offered *BRCA1* and *BRCA2* testing via telephone contact with the study co-ordinator but no formal pre-test genetic counseling to women with a recent diagnosis of epithelial ovarian cancer. Genetic testing revealed a frameshift *BRCA1* mutation, previously reported in the literature as a pathogenic variant causing a high risk of breast and ovarian cancer. The patient was referred for genetic counseling, at which point she asked about the implications of the *BRCA1* mutation on ovarian cancer prognosis, breast cancer risk management and the risk for her first degree relatives. Genetic counselor-initiated discussion included the broader implications for relatives and the psychosocial impact of learning about *BRCA1* mutation status. The patient had an excellent partial response to neo-adjuvant chemotherapy (Carboplatin and Paclitaxel) with no residual disease after total abdominal hysterectomy and no evidence of disease after six months of adjuvant chemotherapy. The patient was interested in accessing risk reducing breast surgery and after an initial discussion 17 months post ovarian cancer diagnosis, she requested follow up in the genetics clinic 2 years and 10 months post diagnosis for a further discussion to weigh up the risks and potential benefits. The patient articulated her motivations for asking about risk reducing breast surgery as wanting to stay free from cancer for as many years as possible. She was already familiar with information about ovarian cancer prognosis in general and her own excellent response to treatment from appointments with her oncology team.

## Breast Cancer Risk in *BRCA1/BRCA2* Carriers after Ovarian Cancer Diagnosis

A small number of studies have indicated that breast cancer risk is lower in *BRCA1/BRCA2* mutation carriers after an ovarian cancer diagnosis, compared to unaffected mutation carriers (Domchek et al. 2013; Gangi et al. 2014; McGee et al. 2017; Vencken et al. 2013). However, data on breast cancer risk in *BRCA1/BRCA2* carriers after ovarian cancer are limited as a result of the poor overall survival. As part of the study by Vencken et al., 79 women with *BRCA1*/*BRCA2*-associated (mostly advanced stage) ovarian cancer but no personal history of breast cancer were followed over 10 years. The women were calculated as having a lower 5-year risk of primary breast cancer of 6%, compared to 16% for the unaffected mutation carrier controls. The 10-year breast cancer risk in women with *BRCA1/BRCA2*-associated ovarian cancer was 11%, compared to 28% in the control group (mortality rates at five and ten years were 33% and 61%, respectively).

Similarly, Domchek et al. (2013) studied 164 *BRCA1/BRCA2* mutation carriers with epithelial ovarian cancer to assess metachronous breast cancer risk. Among the 164 participants, 18 breast cancers were diagnosed, but no breast cancer related deaths occurred (Domchek et al. 2013). Based on their five and ten year figures for breast cancer free survival and overall survival after ovarian cancer, the authors suggest non-surgical management of breast cancer risk in women with a *BRCA1/BRCA2*-associated ovarian cancer. McGee et al. (2017) also found a lower than expected breast cancer incidence in their study cohort of 509 *BRCA1/BRCA2* mutation carriers followed up to 20 years (mean 6.9 years) after ovarian cancer diagnosis. During the follow up period, 203 women died of ovarian cancer, but only 20 women developed breast cancer. Of the four cancer deaths amongst these 20 women, two died from breast cancer and two from recurrent ovarian cancer. The aim of the study by McGee et al. was to estimate breast cancer risk and all-cause mortality after ovarian cancer in *BRCA1/BRCA2* carriers in order to simulate the impact of providing MRI or risk-reducing mastectomy subsequent to ovarian cancer diagnosis. Their simulation reported a less than 1% reduction in the chance of dying (of all causes) before age 80 due to breast MRI screening and a less than 2% reduction due to risk-reducing mastectomy. In the absence of a consensus on the minimum expected benefit to validate MRI or mastectomy, the authors suggest based on their data that these options should be offered to all *BRCA1/BRCA2* carriers with Stage I/II ovarian cancer, but only offered to those with Stage III/IV ovarian cancer diagnosed before age 50 or at least 10 years post ovarian cancer diagnosis without recurrence.

Further evidence of a reduced breast cancer risk in *BRCA1/BRCA2* carriers after ovarian cancer compared to controls was provided by a database review conducted by Gangi et al. (2014). Of 135 mutation carriers followed up between 1998 and 2012, 12 (8.9%) developed breast cancer and seven of these tumors were detected at mammography. All were early-stage breast cancer diagnoses (stages 0-II). At a median follow-up of 6.3 years, four of the 12 women (33.3%) died of recurrent ovarian cancer after a diagnosis of breast cancer, whilst overall survival of the 135 women was 17% (Gangi et al. 2014).

The majority of epithelial ovarian cancers initially respond well to platinum-based chemotherapy, but long term survival is limited by recurrence of tumor cells which develop drug resistance (Bookman et al. 2009). International consensus guidelines state that the mainstay of ovarian cancer treatment involves surgery aiming at complete resection, followed by platinum and taxane-based chemotherapy (du Bois et al. 2009). The most important prognostic factor appears to be tumor burden at diagnosis, classified by FIGO stage I-IV, followed by completeness of cytoreductive surgery, exemplified in a study of 3126 women with ovarian cancer where a subset of 63 women had FIGO stage IV ovarian cancer with no macroscopic residual tumor after surgery (du Bois et al. 2009). Approximately 25% of women in this subset had progression-free survival five years later. A similar likelihood of progression-free survival was shown after five years in a large cohort of women with FIGO stage III or IV epithelial ovarian cancer (Bookman et al. 2009) but these studies did not take *BRCA1/BRCA2* mutation status into account.

## Germline *BRCA1/BRCA2* Mutations and Ovarian Cancer Treatment Outcomes

Germline *BRCA1*/*BRCA2* mutation status impacts on treatment outcomes in ovarian cancer, as shown in a study of 1001 women with epithelial ovarian cancer which found that 14.1% carried a germline mutation in *BRCA1* or *BRCA2* (Alsop et al. 2012). In this study, mutation carriers were more often diagnosed at an advanced stage of disease, but also had better treatment outcomes; after adjustment for age, stage and extent of surgical resection, *BRCA1* and *BRCA2* mutation carriers had improved 5-year progression-free survival and overall survival. The best 5-year progression-free survival of approximately 50% was observed in women with a germline *BRCA1* mutation with no residual tumor after surgery. A favorable effect on 5-year survival in *BRCA1/BRCA2* carriers with ovarian cancers was also seen in a pooled analysis of 26 observational ovarian cancer survival studies (Bolton et al. 2012). However, other studies have produced conflicting results, indicating no advantage in 5-year overall survival in *BRCA1* carriers (Yang et al. 2011). There is evidence suggesting that any short to medium term survival advantage may not translate into significantly higher long-term survival (Candido-dos-Reis et al. 2015; McLaughlin et al. 2012), although substantial data on this are lacking.

## Case Report: Genetic Counseling Provision

We provided an approximate 5-year risk of breast cancer as 10%, based on her current age and *BRCA1* status (Mavaddat et al. 2013). Based on individual factors, we then discussed how her absolute risk of breast cancer over the five years following diagnosis may be lower than this, due to evidence suggesting a reduction in breast cancer risk after ovarian cancer diagnosis in *BRCA1* carriers. We also mentioned a potentially protective cytotoxic effect of chemotherapy on any early breast cancer cells. We were unaware if the patient had been recruited to the treatment or control arm of a PARP inhibitor trial, but the possibility of a similar cytotoxic impact on very early breast cancer from PARP inhibitors was highlighted. The patient was somewhat reassured by having regular chest CT scans arranged for all participants of the PARP inhibitor trial, as this might increase the chance of picking up a breast tumor at an early stage, alongside annual breast screening.

We discussed the associated risks and benefits of risk reducing breast surgery and reconstruction. Information on risks included how surgery may not always go as planned, how women are not always pleased with the look and feel of reconstructed breasts and there can be problems with infection, poor wound healing and chronic pain. We also provided information on average timescales, as even when the surgery and recovery go well, the process still takes many months, with a reduction in quality of life during this period. We balanced this with the obvious advantage of reducing the lifetime breast cancer risk significantly, to less than 5%, with subsequent breast screening not required due to the minimal amount of residual breast tissue.

We spent some time weighing the approximate 10% risk of breast cancer over five years with an estimated 50–75% chance of recurrent ovarian cancer over the same time period. The aim of providing information in this context was to increase the patient’s knowledge about breast cancer risk, as well as potentially reducing breast cancer risk perception and anxiety during the five year period of ovarian cancer treatment and active follow up. Genetic counseling interventions involved presenting the information in verbal and visual format (numerical and graphical), checking understanding and providing a designated time and space to focus on current concerns. The patient did not appear anxious or distressed by the information provided, but did express gratitude for the opportunity to ask questions in a review appointment. Towards the end of the session, she asked at what stage it would be advisable to review the question about breast cancer risk management and the suggestion of a review on request five years after diagnosis was mutually agreed.

## Case Report: Genetic Counseling Challenges

If and when risk-reducing breast surgery in *BRCA1/BRCA2* carriers with a previous diagnosis of ovarian cancer is appropriate is not easily answered by current research evidence. This is reflected by guidelines on breast cancer risk management in *BRCA1/BRCA2* carriers, which do not give clear recommendations for women with a previous diagnosis of ovarian cancer, but support discussion of the option of risk-reducing breast surgery with women on a case-by-case basis (NCCN, 2016). Contraindications to risk-reducing breast surgery are stated in the UK NICE guidelines as co-morbidities that would considerably increase the risks of surgery or that cause limited life expectancy (NICE 2013). Uncertainty about recurrence of ovarian cancer featured prominently in the genetic counseling we provided. In discussing this, we were aware of blurring boundaries between the role of clinical genetics and oncology. Fortunately, we had support and close liaison with the patient’s oncology team and this case highlighted the benefits of the multi-disciplinary approach.

The chance of surviving ovarian cancer increases with the number of recurrence-free years post diagnosis (Narod 2016). The figures used in our genetic counseling case were mainly risks over a five-year period. Five-year risks are often used in genetic counseling for *BRCA1/BRCA2* carriers, due to the available data from relevant research studies. Five-year risks were also chosen for genetic counseling in this case in order to answer the patient’s questions addressing risk management in the short- to medium-term. In our case report, a fuller discussion on the potential risks and benefits of risk-reducing breast surgery took place almost three years after ovarian cancer diagnosis without signs of recurrence. In this situation, it may be more appropriate to consider the impact on the risk/benefit ratio at the ten-year mark. Ten years after ovarian cancer diagnosis there is less uncertainty about the chance of relapse, which is significantly reduced (Narod 2016). However, ten years after ovarian cancer diagnosis, our patient will be age 64 years, at which point her remaining lifetime risk of breast cancer will be smaller and the risks associated with any surgery will be increased. From a genetic counselor’s perspective, discussing ten year risks in the initial years after a diagnosis of ovarian cancer may not best answer their patients’ questions, given the poor overall survival and need for a shorter-term plan for breast cancer risk management.

When providing genetic counseling to women with ovarian cancer and a recently identified *BRCA1/BRCA2* mutation, genetic counselors have breast cancer risk management on their agenda. Who raises this issue and when it is raised during the consultation depends on many factors, which may include the patient’s pre-test understanding of *BRCA1/BRCA2* mutation risks, family history and response to ovarian cancer treatment. In the case described here, information on breast cancer risk management options, including risk-reducing strategies, were presented in general terms. In this way, risk-reducing surgery was raised as having been proven to reduce breast cancer risk in *BRCA1/BRCA2* carriers, but also as something not usually considered when the focus is on ovarian cancer treatment. For women who have an excellent response to treatment and are well two to three years later, there is potential for a mismatch between what a genetic counselor perceives as a remaining focus on ovarian cancer and how a patient views this, whilst being told there are no signs of relapse at regular oncology reviews. For many women, who are not interested in risk-reducing mastectomy, further genetic counseling to address this time-sensitive issue may not be required. For those interested in risk-reducing mastectomy, review is needed to provide updated information on the potential risks and benefits, as well to provide psychosocial support in decision making.

Part of the genetic counseling acknowledged the importance of hope and optimism as strategies for coping with uncertainty. There is evidence to show that fear of ovarian cancer recurrence is linked to reduced levels of hope (Ozga et al. 2015) and that hope is an important factor for women in enabling them to face the threat of ovarian cancer recurrence after completion of first-line treatment (Reb 2007). Hope and optimism have also been found as facilitating adaptation to an increased familial breast cancer risk, when this has been studied in women from high-risk families with and without a *BRCA1/BRCA2* mutation (Heiniger et al. 2015). We tried to sustain hope by checking how much information the patient wanted about risks of ovarian cancer recurrence, whilst acknowledging the limitations of presenting data that cannot accurately predict an individual’s future. Checking information preferences, alongside an awareness of and empathy for the potential impact of the information provided are communication skills integral to genetic counseling.

## Considerations for Genetic Counseling Practice

Following the identification of a *BRCA1/BRCA2* mutation shortly after ovarian cancer diagnosis, the initial focus is often placed on treatment implications for the woman and risks to her relatives. This report highlights the genetic counseling needs of the index case and shows how these needs can change over time. Genetic counseling for women with ongoing questions about breast cancer risk management is time sensitive and is influenced by the number of years since ovarian cancer diagnosis. Who initiates genetic counseling review will depend on various factors, including if the clinical genetics center has a regular recall system for *BRCA1/BRCA2* carriers. In the absence of systematic follow up, genetic counselors should seek to empower patients at every opportunity, so that they willingly seek further genetic counseling input as required.

The provision of genetic counseling described was aided by a supportive relationship with oncology colleagues. As genetic testing becomes integrated into the routine management of individuals with ovarian cancer, more women with a germline *BRCA1/BRCA2* mutation will be identified. Open and supportive dialogue between clinical genetics and oncology health professionals is likely to have patient benefits. For women with ovarian cancer and a *BRCA1/BRCA2* germline mutation who are interested in risk-reducing breast surgery, realistic information should be presented in an individualized way, in order to facilitate women making decisions about their own life and body. Individual factors in this situation will include tumor stage, time since diagnosis, current health, age, patient breast cancer risk perception and risk management preferences. This involves a careful balance of providing complex risk information whilst taking into account the information and emotional needs of the individual. Clinical genetics professionals are well placed to provide this service. Qualitative research to evaluate how women adapt to and view *BRCA1/BRCA2*-related breast cancer risk after ovarian cancer could further inform genetic counseling practice. Longer term quantitative studies are also needed to provide information about breast cancer risk to women with a *BRCA1/BRCA2* mutation who are disease-free more than five years after treatment for ovarian cancer.
